# Quantitative patient‐specific quality assurance prediction using MLC mean leaf gap and PTV volume

**DOI:** 10.1002/acm2.70146

**Published:** 2025-07-13

**Authors:** Caroline M. Colbert, Eric C. Ford, Minsun Kim

**Affiliations:** ^1^ Department of Radiation Oncology University of Washington and Fred Hutch Cancer Center Seattle Washington USA

**Keywords:** PSQA, plan complexity, treatment planning

## Abstract

Rigorous patient‐specific quality assurance (PSQA) is essential to radiation therapy safety. As logfile‐based PSQA gains adoption, quantitative methods to select certain high‐complexity treatment plans for additional measurement‐based PSQA can help to ensure a comprehensive QA program. We propose a simple metric to predict PSQA measurement results for stereotactic body radiation therapy (SBRT)–volumetric modulated arc therapy (VMAT) plans based on historical QA results, integrated into the treatment planning process for the early detection of potential issues. This method can be used to screen treatment plans for measurement‐based QA, saving time while maintaining safety standards. We identified 46 SBRT–VMAT plans for C‐arm LINACs and 18 plans for a ring gantry LINAC that underwent PSQA with gamma analysis thresholds of 3%, 2 mm in our clinic. We developed a script to compute the MLC mean leaf gap (MLG) width as a proxy for treatment plan deliverability, which can be run during the planning process. We analyzed the correlation of MLG with gamma pass rates and performed a receiver operating characteristic (ROC) analysis of its performance as a binary predictor of QA measurement results. We found a significant positive correlation between gamma pass rate and overall MLG (*R* ^2 ^= 0.39, *p* < 0.001) for SBRT–VMAT plans. A criterion of MLG < 2.02 cm predicts PSQA failure with a sensitivity of 0.87 and a specificity of 0.74 for C‐arm LINAC plans. For ring gantry plans, MLG < 2.13 cm showed a sensitivity of 1.00 and specificity of 0.60. A simple descriptive metric based on historical QA results can be used to screen treatment plans that might need further analysis with logfile‐ or measurement‐based PSQA.

## 1 Introduction

The commercial availability of logfile‐based patient‐specific quality assurance (PSQA) methods has challenged the prevailing paradigm of measurement‐based PSQA.^1^ Many previous studies have attempted to predict PSQA measurement results using quantitative radiotherapy plan features. Such a method may provide justification for alternative PSQA approaches for plans at low risk of failure. As logfile‐based PSQA gains adoption, measurement‐based PSQA may still be preferred for certain complex plans. Clinical PSQA history can be used to identify metrics to screen plans for additional measurement‐based QA, while introducing minimal additional overhead in treatment plan preparation.

Prior studies have investigated a broad range of plan complexity metrics with the aim of predicting PSQA failure.^2^, ^3^ Chiavassa et al. divide these metrics into three groups.^3^ One group of metrics describes the complexity of the fluence map, including texture features like the maximum intensity ratio. Other metrics, such as modulation factor or variation in leaf travel or gantry speed, relate to plan deliverability. The third category captures MLC leaf gaps or field areas to describe the prevalence of challenging MLC configurations, and therefore the uncertainty of dose computation.^3^ Following a broad preliminary analysis of many plan complexity metrics, we chose to focus on metrics in the third category relating to MLC leaf gap width. Leaf gap width is influenced by several factors in treatment planning, including target shape and volume, and modulation factor. As dose computation accuracy is heavily dependent on the accuracy of the leaf‐tip model,^4^, ^5^, ^6^ correlation between MLC leaf gap metrics and PSQA results may indicate that results are primarily beam model‐limited.

Previous studies have aimed to reduce QA workload by predicting which plans will pass with sufficiently high confidence as to obviate the need for physical beam delivery prior to the first fraction.^7^, ^8^, ^9^, ^10^, ^11^ In contrast, we aim to identify plans that can safely undergo logfile analysis of a full physical beam delivery, but without a 3D diode measurement. As radiation oncology clinics increasingly turn toward plan delivery logfile verification for PSQA,^12^ ^–^ ^15^ we aim to identify individual treatment planning metrics that can be used to screen plans for an additional measurement‐based analysis in addition to logfile‐based QA.

## 2 METHODS

We collected 23 clinical SBRT–VMAT treatment plans created between 2022 and 2023 in our clinic for delivery on an Elekta Agility™ LINAC that showed a gamma pass rate less than 90%. PSQA was performed for all plans on an SNC ArcCheck (Sun Nuclear Corp., Melbourne, FL, USA) and analyzed with a gamma criterion of 3%/2 mm, a 10% dose threshold, and global normalization (SNC Patient 8.4.1), in accordance with the recommendations of AAPM Task Group 218.^16^ We selected an equal number of plans with gamma pass rates above the 90% passing threshold for the same body sites, sampled randomly over all delivered plans. All treatment plans were created by certified medical dosimetrists in RayStation 8B (RaySearch Laboratories, Stockholm, Sweden). Similarly, we collected eight SBRT–VMAT treatments planned in the Varian Ethos 2.1 (Varian Medical Systems, Palo Alto, CA, USA) treatment planning system (TPS) in 2023 and 2024 with gamma pass rates less than 90%, and 10 randomly selected plans with gamma pass rates above 90%.

We evaluated the overall MLC mean leaf gap (MLG) for each plan, defined as the unweighted mean distance between in‐field opposing leaf pairs over all VMAT segments, using an in‐house, Python 2.7 scripting protocol within RayStation 8B. Varian Ethos treatment plans were exported to RayStation to compute MLG. The planning target volume (PTV) size of all targets was recorded, as well as an Ethos‐specific small field warning.

Statistical analysis was performed in OriginPro (9.8.0.200). The Shapiro–Wilk test was used to evaluate normality (*p* = 0.05) for descriptive and comparative statistics. Normally distributed data are reported as mean ± standard deviation; otherwise, data are reported as median (inter‐quartile range), and nonparametric tests are used. Linear regression analysis was used to evaluate the correlation of plan metrics with gamma pass rate. ROC analysis was used to evaluate plan metrics as predictors of binary PSQA measurement results (pass vs. fail).

## 3 RESULTS

### 3.1 C‐arm LINACs

**FIGURE 1:**
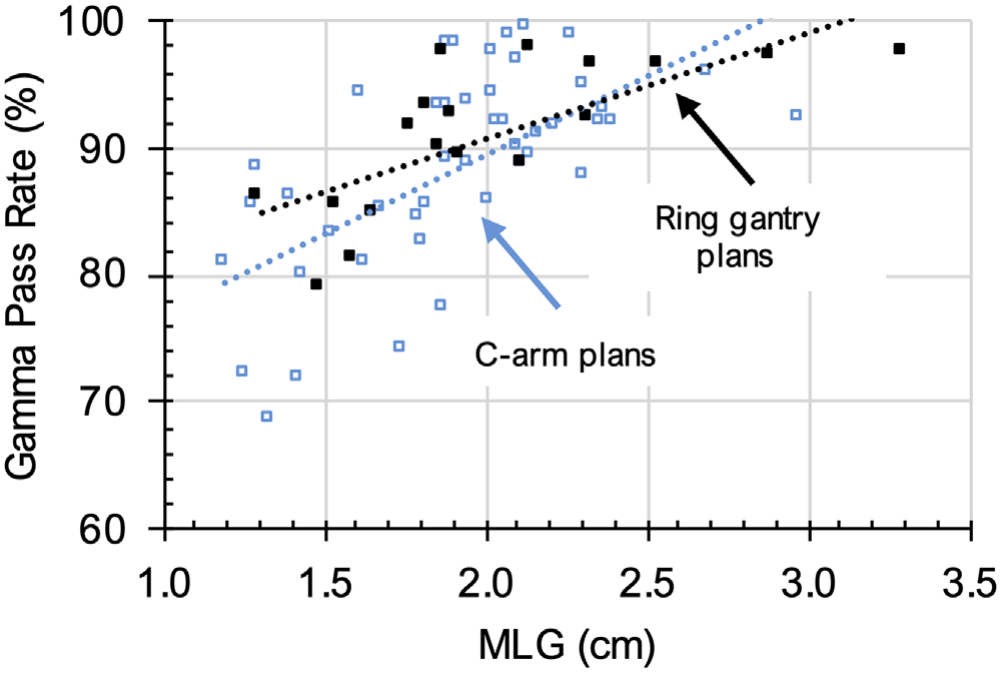
MLG vs. gamma pass rate. We found a significant correlation between MLG and gamma pass rate for C‐arm LINAC plans (*R* ^2^ = 0.39, *p* < 0.001) and ring gantry plans (*R* ^2^ = 0.53, *p* < 0.001) on linear regression analysis. Blue, C‐arm LINAC plans; black, ring gantry LINAC plans.

All 23 SBRT–VMAT plans with gamma pass rates less than 90% were planned for targets in the lung. In addition, 23 lung SBRT–VMAT plans were randomly selected, which passed PSQA with gamma pass rates > 90%. Mean MLG among all 46 plans was 1.92 ± 0.38 cm. On linear regression analysis, MLG was correlated with gamma pass rate (*R* ^2 ^= 0.39, *p* < 0.001, Figure 1). On ROC analysis, MLG showed an area under the curve (AUC) of 0.87 (95% CI 0.77, 0.98) as a predictor of binary PSQA result. A criterion of 2.02 cm showed a sensitivity of 0.87 and a specificity of 0.74 for the prediction of QA measurement results. We found a mean MLG of 1.69 ± 0.32 and 2.15 ± 0.29 cm among failing and passing plans, respectively.

Among these 46 plans, we found a median PTV volume of 18.3 cc with an inter‐quartile range, IQR: 9.2, 31.7. Target volume varied widely in the cohort: PTV volume ranged from 4.5 to 151 cc. Plans with a gamma pass rate < 90% showed a median PTV volume of 9.5 cc (IQR 6.4, 16.9), while the median PTV volume for passing plans was 29.4 cc (IQR 19.4, 46.4). Log‐transformed PTV volume correlated with gamma pass rate (*R* ^2^ = 0.42, *p* < 0.001) and proved comparable to the MLC leaf gap metrics as a predictor of PSQA result for C‐arm LINAC plans (AUC 0.88, 95% CI 0.79, 0.98).

### 3.2 Varian Ethos

The eight SBRT–VMAT treatments planned in Ethos 2.1 with gamma pass rates less than 90% targeted sites in the lung, pelvis, and bony anatomy. Ten SBRT–VMAT treatments planned on Ethos with gamma pass rates greater than 90% were chosen from the same range of sites. Taken together, these 18 plans showed a mean MLG of 2.02 ± 0.51 cm, with mean values of 1.69 ± 0.26 and 2.29 ± 0.50 cm for failing and passing plans, respectively. MLG showed a comparable AUC for prediction of measurement‐based QA outcome on the ring gantry LINAC (0.88, 95% CI 0.71, 1.04) and was also closely correlated with gamma pass rate (*R* ^2 ^= 0.53, *p* < 0.001, Figure 1). A criterion of MLG < 2.13 cm maximized sensitivity at 1.00, and corresponded to a specificity of 0.60, incorrectly predicting QA failures for four plans that ultimately passed PSQA. Additional MLC leaf gap metrics, which performed well for Varian Ethos plans, are reported in Appendix 1.

Varian Ethos plans also showed a divergence in PSQA results by target volume: plans with low gamma pass rates showed a median PTV volume of 12.3 cc (IQR 8.6, 14.8), while passing plans showed a median PTV volume of 52 cc (25.6, 68.8). Log‐transformed PTV volume was also highly predictive of PSQA result, with an AUC of 0.92 (95% CI 0.77–1.07). The Ethos TPS showed a small field alert for eight of the eighteen SBRT–VMAT plans. Although a nonparametric Mann–Whitney test found a significantly lower median gamma pass rate among plans flagged with a small field alert (85.8 [82.1, 92.9]) than those without (94.5 [89.8, 97.2], *p* = 0.046), ROC analysis showed gamma pass rate to be a weak predictor of the presence of a small field warning, with an AUC of 0.788 (95% CI 0.568, 1.01) and disagreement of results in six cases.

## 4 DISCUSSION

Prior studies that have attempted to discriminate IMRT QA results a *posteriori* often use multi‐parametric models trained on a wide range of plan metrics extracted from over 200 plans, few of which fail PSQA.^7^, ^11^ These models are ultimately highly specific to individual institutions’ historical QA results.^7^, ^8^, ^10^ As an exception, in a multi‐institutional validation study, Valdes et al. report that a model trained to predict PSQA results to within 3% using a diode device at one institution was then able to predict portal dosimetry measurement results of many plans (120 out of 139) to within 3.5% at a second institution.^9^ Although this points to the cross‐institutional applicability of some models, many clinics would likely prefer a simpler solution validated against their own institutional data.

We identified several metrics predictive of PSQA measurement results, including log‐transformed target volume, mean MLC leaf gap, and others reported in supplementary materials. These treatment plan metrics are closely related, and each would be acceptable to screen plans for measurement‐based or logfile‐only PSQA. Although log‐transformed PTV volume does not require a scripting solution to evaluate, MLG has the benefit of decoupling predicted results from target shape. For example, elongated target volumes can have a large volume but are best suited by a plan with narrow MLC leaf gaps. The plans in our dataset delivered on the Elekta Agility and Varian Ethos were created in different treatment planning systems with different dose computation algorithms and LINAC head models with substantially different MLC designs. That these metrics worked well in both vendor ecosystems indicates that the challenges related to delivering highly modulated plans to small targets are not specific to a single LINAC design, beam model, or TPS configuration. However, the present study is insufficient to demonstrate the generalizability of the specific data conclusion of this analysis to other sites, such as head and neck, to plans with HDMLC, or other complex techniques. A broader multi‐institutional study would be required to determine whether the pattern observed in the data holds true at other institutions. However, our primary aim is to demonstrate the utility of the overall method we used to identify a screening method for measurement‐based QA based on our historic QA records.

Identification of a single metric to predict PSQA results represents a simpler solution than those proposed in previous works. Wall et al. report an AUC of 0.95 when predicting ion chamber dose errors > 3% in a Mobius QA phantom, using multiple features including jaw aperture area, edge metric, MU factor, and small aperture score.^17^ Xu et al. used the agreement of TPS‐ and secondary Monte Carlo‐computed dose to predict ArcCheck PSQA failure (< 90% gamma pass rate, 3%/3 mm) with an AUC of 0.95.^11^ Han et al. report an AUC of 0.89 to predict PSQA failure (< 90% gamma pass rate, 3%/2 mm) using a model including plan complexity metrics, dosiomics features and a deep learning‐based PSQA score.^7^ Using the mean MLC leaf gap, we report an AUC of 0.87 and 0.88 for Elekta C‐arm LINAC plans and Varian Ethos plans, respectively. In contrast to these studies, a notable limitation of this work is its narrow focus on SBRT–VMAT plans, particularly in the lung. In practice, this selection process limits the clinical applicability of our simple benchmarks to similar SBRT–VMAT plans. In the absence of the generalizable methods published in prior works, establishing body site‐specific benchmarks will be necessary for prospective screening.

PSQA measurements with low gamma pass rates can indicate errors in plan delivery or modeling in the TPS. However, diode array‐based PSQA is known to have significant limitations, most notably the well‐documented inability of gamma analysis to detect introduced errors.^4^, ^18^, ^19^, ^20^, ^21^ Large dose differences caused by introduced plan delivery errors can occur in cases with high gamma pass rates.^19^ Such errors can significantly degrade TCP and NTCP in clinical plans.^22^ Nelms et al. report that systematic errors, which went undetected on gamma analysis, were identifiable on delivery fidelity analyses like those performed by our logfile QA software (Mobius FX, Mobius Medical Systems, Houston, TX). Despite the known weaknesses of gamma analysis, gamma pass rate < 90% is the primary endpoint of this study. Clinics that wish to screen for complex plans for measurement‐based QA aim to ensure that all clinically treated plans would pass a gamma analysis, regardless of that method's weaknesses. In the absence of an alternate consensus reference standard, future studies of virtual QA methods will continue to use gamma analysis as a benchmark.

A balanced discussion requires acknowledgement of the limitations of logfile‐based PSQA methods: their discriminatory power is highly dependent on plan type and may vary with factors such as disease site, dose gradient, off‐axis fields, and so forth. As a result, as logfile analysis gains adoption, it is necessary to compare logfile‐based QA measurements against existing QA methods using test plans that comprehensively reflect the range of plan types, and some clinics may prefer to continue measurement‐based PSQA for complex plans. Simple plan metrics can be used to screen plans for measurement‐based QA, while introducing minimal additional overhead in treatment plan preparation.

Institutional PSQA history can be used to identify individual plan complexity metrics, which can serve as predictors of PSQA results across platforms. As the clinical implementation of logfile analysis grows, simple interpretable screening criteria for measurement‐based PSQA can help to ensure a comprehensive QA program.

## AUTHOR CONTRIBUTIONS

Conception and development of the work: all authors; scripting, data collection, and analysis: Caroline M. Colbert; initial manuscript preparation: Caroline M. Colbert; critical review and final approval of the manuscript: all authors.

## CONFLICT OF INTEREST STATEMENT

The authors declare no conflicts of interest.

## Supporting information



Supporting Information

## Data Availability

The data that support the findings of this study are available from the corresponding author upon request.
