# Correction to: “Quantitative patient‐specific quality assurance prediction using MLC mean leaf gap and PTV volume”

**DOI:** 10.1002/acm2.70412

**Published:** 2025-12-15

Mills MD. Quantitative patient‐specific quality assurance prediction using MLC mean leaf gap and PTV volume. *J Appl Clin Med Phys*. 2025;e70412. https://doi.org/10.1002/acm2.70146

The original article was incorrectly published as an Announcement. The article should have been published as a Research Article. The online version of this article has been corrected accordingly.

