# Supplementary material for: Quantitative patient‐specific quality assurance prediction using MLC mean leaf gap and PTV volume
Source: J Appl Clin Med Phys. 2025 Jul 13;26(7):e70146. doi: 10.1002/acm2.70146 (PMC12256692; doi:10.1002/acm2.70146)
Supplement: Supplementary file 1 — Supporting Information [file ACM2-26-e70146-s001.docx]

Appendix 1: Additional analysis of MLC leaf gap subsets.

The mean leaf gap (MLG) of the smallest and largest 10%, 25%, and 50% of all open leaf pair gaps (MLG-S10, MLG-S25, MLG-S50; MLG-L10, MLG-L25, MLG-L50) were analyzed for prediction of PSQA measurement results. MLG-L25 showed an AUC of 0.88 for C-arm LINAC plans, offering little additional predictive power relative to overall MLG. For ring gantry LINAC plans, MLG-L25 was the best predictor of QA failure, with an AUC of 0.93. A criterion of MLG-L25 < 3.5 cm maximized specificity at 0.70, incorrectly predicting QA failures for three plans that ultimately passed PSQA.

| Metric | AUC (95% CI) | Criterion | Sensitivity | Specificity |
| --- | --- | --- | --- | --- |
| SBRT-VMAT Plans for C-arm LINAC | | | | |
| MLG | 0.87 (0.77, 0.98) | 2.02 | 0.87 | 0.74 |
| MLG-L50 | 0.88 (0.78, 0.98) | 2.67 | 0.87 | 0.83 |
| MLG-L25 | 0.88 (0.79, 0.98) | 3.20 | 0.87 | 0.70 |
| MLG-L10 | 0.87 (0.77, 0.97) | 3.73 | 0.87 | 0.61 |
| MLG-S10 | 0.65 (0.49, 0.82) | 0.60 | 0.87 | 0.09 |
| MLG-S25 | 0.68 (0.53, 0.84) | 0.79 | 0.87 | 0.30 |
| MLG-S50 | 0.78 (0.64, 0.92) | 1.20 | 0.87 | 0.43 |
| SBRT-VMAT Plans for Ring Gantry LINAC | | | | |
| MLG | 0.88 (0.71, 1.04) | 2.13 | 1.0 | 0.60 |
| MLG-L50 | 0.91 (0.78, 1.04) | 3.03 | 1.00 | 0.60 |
| MLG-L25 | 0.93 (0.81, 1.04) | 3.50 | 1.00 | 0.70 |
| MLG-L10 | 0.90 (0.75, 1.05) | 4.04 | 1.00 | 0.70 |
| MLG-S10 | 0.70 (0.45, 0.95) | 0.67 | 1.00 | 0.40 |
| MLG-S25 | 0.79 (0.57, 1.01) | 0.88 | 1.00 | 0.50 |
| MLG-S50 | 0.79 (0.57, 1.01) | 1.23 | 1.00 | 0.50 |

Table 1. ROC analysis of MLG metrics applied to 46 SBRT-VMAT treatment plans for C-arm LINACs and 18 SBRT-VMAT plans for a ring gantry LINAC. MLG-LX represents the mean MLC leaf gap of the largest X percent of MLC leaf gaps in the treatment plan. MLG-SX represents the mean MLC leaf gap of the smallest X percent.
